# 367. Role of Conventional Biomarker for Prediction of Chest CT-confirmed COVID-19 Pneumonia

**DOI:** 10.1093/ofid/ofab466.568

**Published:** 2021-12-04

**Authors:** Monprach Harnphadungkit, Taweegrit Siripongboonsitti

**Affiliations:** Chulabhorn Hospital, HRH Princess Chulabhorn College of Medical Science, Chulabhorn Royal Acedemy, Bangkok, Thailand, Lak si, Krung Thep, Thailand

## Abstract

**Background:**

The coronavirus disease 2019 (COVID-19) has a wide range of severity. Chest computed tomography (CT) had high sensitivity and specificity to identify COVID-19 pneumonia. However, chest CT was not available in almost all hospitals in pandemic settings, including developed countries. This study is to evaluate the potential role of conventional inflammatory biomarkers to predict COVID-19 pneumonia.

**Methods:**

All 155 RT-PCR-confirmed COVID-19 patients were evaluated for pneumonia by chest CT from April 10, 2021 to May 3, 2021 in the outpatient unit, a Thai university hospital. The inflammatory biomarkers were evaluated the sensitivity, specificity, LR+, LR-, and ROC to predict COVID-19 pneumonia.

**Results:**

Of all 155 patients, pneumonia was diagnosed by chest CT in 117 patients. The pneumonia patients had a median (IQR) age of 38 (30, 55) years old. The BMI was higher in pneumonia than mild illness in 25.5 (22.0, 29.5) and 22.9 (19.4, 26.9) kg/m^2^, respectively (p=0.031). In univariate analysis, serum high-sensitivity C-reactive protein (hsCRP), lactate dehydrogenase (LDH), ferritin, total lymphocyte count (TLC), and albumin were associated with pneumonia, but the only hsCRP demonstrated association by multivariate analysis. The area under the ROC curves (AUC) was 0.82, 0.74, 0.68, 0.38, and 0.37 in hsCRP, LDH, ferritin, TLC, and albumin, respectively. The optimal cut-off level for CRP to diagnose COVID-19 pneumonia was 2.00 mg/L given sensitivity, specificity, LR+, LR- of 81.9%, 70.3%, 2.75, and 0.26 respectively (Figure 1 and Table 1).

ROC Curve of hsCRP to Diagnose of COVID-19 Pneumonia

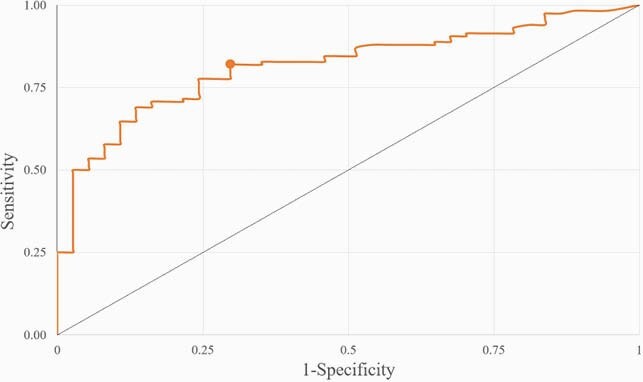

This figure shows ROC curve for hsCRP to diagnose of chest CT-confirmed COVID-19 pneumonia. The area under the ROC curve is 0.82. The optimal cut-off value for hsCRP is 2.00 given sensitivity of 81.9% and specificity of 70.3%.

**Conclusion:**

The hsCRP was the conventional biomarker that had an excellent performance in predicting COVID-19 pneumonia lead to early anti-SARS-CoV-2 treatment. This study demonstrated the potential role of hsCRP combined with clinical assessment in negative chest X-rays to replace chest CT in a high burden COVID-19 country during pandemic situations.

**Disclosures:**

**All Authors**: No reported disclosures

**Table 1.** Demonstrated sensitivity, specificity, LR+, and LR- for each specific cut-off value of hsCRP

Cut-off for hsCRP (≥ mg/L)	Sensitivity (%)	Specificity (%)	LR+	LR-
1.90	81.9	64.9	2.33	0.28
1.95	81.9	67.6	2.53	0.27
2.00*	81.9	70.3	2.75	0.26
2.05	80.2	70.3	2.70	0.28
2.10	80.2	70.3	2.70	0.28

*Indicated optimal cut-off value for hsCRP to predict chest CT-confirmed pneumonia.

